# Study protocol for leaving care—A comparison study of implementation, change mechanisms and effectiveness of transition services for youth

**DOI:** 10.1371/journal.pone.0293952

**Published:** 2024-02-08

**Authors:** Therése Skoog, Martin Bergström, Matilda Karlsson, Tina M. Olsson

**Affiliations:** 1 Department of Psychology, University of Gothenburg, Göteborg, Sweden; 2 School of Social Work, Lund University, Lund, Sweden; 3 Department of Social Work, University of Gothenburg, Göteborg, Sweden; 4 Department of Social Work, School of Health and Welfare, Jönköping University, Jönköping, Sweden; PLOS: Public Library of Science, UNITED KINGDOM

## Abstract

**Introduction:**

Youth placed in out-of-home care is a large and highly vulnerable group at high risk of negative developmental outcomes. Given the size and extent of negative developmental outcomes for youth placed in out-of-home care, interventions to help this vulnerable group navigate successfully towards independent living and promote wellbeing across a spectrum of outcome areas are needed. To date, there is a lack of such interventions, particularly in Sweden. Importing interventions from other societies and cultures is associated with difficulties. The aim of the research project is to implement, test, and evaluate interventions that have been recently developed in Swedish practice to close this gap.

**Methods:**

The project has an ambitious and complex data collection and analysis strategy using qualitative, quantitative, and multiple information methods (hybrid effectiveness-implementation study) over the course of two years. Both the implementation and effectiveness of the interventions will be evaluated. The recently developed My Choice-My Way! leaving care program for youth aged 15+ will be the primary focus of the project and will be compared to usual services.

**Conclusions:**

The project has the potential to offer novel insights into how society can promote wellbeing across a spectrum of outcome areas for the high-risk group of youth transitioning from out-of-home care to independent living. As such, the project will have important implications for both research and practice.

**Trial registration:**

ClinicalTrials.gov Identifier: NCT05813197.

## Introduction

### Background and rationale

This protocol describes an applied Swedish research project on how to promote the well-being across a spectrum of outcome areas for youth transitioning from out-of-home care to independent living.

Youth placed in out-of-home care is a large and highly vulnerable group with high risks of negative, developmental outcomes. About 3–4% of Swedish children are placed in out-of-home care (i.e., care situations where youth live outside of their own home e.g., foster care, group home care, institutional care) at some point and about 1% of children grow-up primarily in out-of-home care [1]. Most of the children placed in out-of-home care are teenagers. Swedish research continually shows that these youth transition to independent living and adulthood with relative disadvantage in all types of health related outcomes (e.g. somatic, mental, and dental; see meticulous review by [2]), educational attainment [3], housing stability [4], early childbearing and reproductive health [5], substance abuse [6], offending [7], exclusion from or weak attachment to the labor market [8], and public welfare dependency [7] compared to their non-placed peers. This negative development pattern is consistent across studies regardless of outcome, choice of comparison group, methodological approach, or location of study.

Given the size and extent of negative developmental outcomes for youth placed in out-of-home care, interventions to help this vulnerable group navigate successfully towards independent living and promote wellbeing across a spectrum of outcome areas are needed. Unlike other countries there is no legislation regarding the provision of transition services for this group in Sweden. Current practice places emphasis on activities which occur prior to placement (e.g., recruitment of foster families) with little to no interventions provided for support of children during ongoing care [9]. None of the strategies for care of children in out-of-home care currently in use in Sweden have been evaluated for their effectiveness (ibid). There is very little research regarding the effectiveness of interventions for transition aged youth. A review [10] concluded that youth aging out of out-of-home care who participate in the programs investigated obtain some productive outcomes but that comparisons with nonparticipant youth and comparisons between interventions could not be made because the studies included in the review were poorly designed. Another review of interventions designed to improve the physical and mental health of children placed in foster care [9], found that outcomes in this group can be improved but only three of 18 specific interventions had more than “some reliable evidence of effectiveness” (p. 7). Only one of these targeted youth themselves. This result was mirrored in a review of transition services for youth leaving out-of-home care which found that Independent Living Services which adapt services to youth needs can have positive results [11].

Across systematic reviews [9, 11, 12] the potential of self-determination interventions to improve outcomes for this population has been highlighted. Self-determination originates from multiple youth-oriented fields [13] where consensus has emerged around self-determination as a developmental protective factor [14]. A growing body of research has affirmed the promotive role of self-determination in positive youth development [13], and in quality-of-life outcomes (including internalizing and externalizing behaviors) for youth [15]. Intervention to enhance self-determination focuses on the intention to make decisions, to direct one’s actions, and to exercise rights and responsibilities, within the context of an individual’s culture, experiences, and aspirations. Experimental tests of interventions based on self-determination theory are few, have small sample sizes, and have not been replicated. Two intervention components have been replicated across self-determination enhancement studies: youth-directed skill-building to develop specific competencies for accomplishing transition-related goals (selecting goals, problem-solving, self-regulation; [16]), and consistency in promoting positive youth attitudes and beliefs around their capacity for self-determination in their lives [17]. Specific tests of mechanism-based mediation are lacking. This means that even though interventions of this type appear to be promising and have some empirical support for their effectiveness, the causal link between specific program components (e.g., goal setting), theoretical constructs (e.g., behavior regulation) and outcome (e.g., educational achievement) has yet to be tested. Increasing our understanding of *how* specific activities impact change processes has the potential of aiding our ability to design effective programs in the future.

#### Linking behavior change techniques (professional practice components) with mechanisms of action (theoretical constructs) and outcome (client level behavior change)

Little is known about how we might put into practice specific activities (i.e., program content) to impact specific change mechanisms (i.e., constructs) in order to impact outcomes for individuals, groups and society. There exists an abundance of behavior change techniques (e.g., goal setting, problem solving; [18, 19] available as well as theoretical models of behavior and behavior change (e.g., self-determination theory, social cognitive theory; [20]) but empirical testing of the links between these two is lacking [19]. Increased understanding of these links is key to informing the development of effective interventions by providing information on the strategies and target mechanisms likely to be effective in specific contexts and among specific populations [21]. A similar shortcoming concerns the link between change mechanisms and outcome. Recent meta-reviews of interventions across a range of contexts and populations have brought to light the limited extent to which primary studies include theory-based measures of the dependent variable, tend not to conduct the appropriate analyses to test intervention change mechanisms, do not provide enough information for secondary analyses of change mechanisms, and do not specify a proposed theory-based mechanism for the effect of the intervention on individual behavior [22–24]. Although primary studies of the effectiveness of interventions test the effects of intervention on outcome, assessment of how a successful intervention changes behavior is lacking (i.e., via a priori planned and appropriately powered mediation analysis; [23]).

#### Implementing promising interventions within human service organizations

In order for effective interventions to produce positive change in individuals, the implementation process within human service organizations (e.g., municipal social services, NGOs) must also be effective. Few programs survive after the initial implementation period to become a sustained, integral part of regular service (for review, see [25]). Organizations could cease the use of the new program entirely or they could significantly alter it in a way that may or may not be desirable given the current context and target group (for a Swedish example see, [26]). The understanding that interventions can fail due to theoretical deficiencies in the intervention itself or shortcomings in the implementation process has led to increased focus on *how* we might systematically carry out implementation efforts in order to improve implementation effectiveness when moving interventions into practice (for a review of implementation theories, models and frameworks see [27]).

Getting to Outcomes (GTO, [28]) is both an implementation model for carrying out intervention activities, and a support intervention aimed at enhancing practitioner capacity. Built on social cognitive theories of behavioral change and implementation science theories such as the Consolidated Framework for Implementation Research [29], developers purport that GTO builds practitioner capacity to perform multiple implementation best practices needed for interventions. Improved performance of these implementation best practices when delivering a specific intervention may improve intervention fidelity, which should result in more positive outcomes for clients. GTO has been applied to several content areas with active involvement of GTO model developers. Although GTO is an open source material this leaves questions as to whether GTO can effectively be used as a general implementation support (i.e., without developer support or involvement) to boost program effectiveness and sustainability.

#### Project novelty

This project is novel in a number of ways. First, this project weaves together several complementary areas of research to meet the needs of the municipal social services and the youth in its care (i.e., implementation science, intervention science, developmental psychology, social work). Second, this research will experimentally test the effects of a locally developed, theoretically and empirically inspired intervention developed based on cutting-edge intervention development theory in collaboration with municipal stakeholders aiming to support an underserved and extremely vulnerable group, one of the municipal social services core populations. Third, this project will experimentally test the extent to which implementation support impacts fidelity, sustainability and client outcomes. Fourth, this research will increase our empirical understanding of how specific behavior change techniques (practice components) are related to theoretical change mechanisms (constructs) and how these constructs in turn are related to youth outcomes. This has the prospect of increasing our scientific knowledge of how intervention development and implementation can be approached in the future. Finally, there are currently no evidence-based and culturally sensitive interventions available to social service professionals for this population and purpose. Our work has the goal of developing our knowledge toward meeting this challenge.

### Objectives

The purpose of this project is to increase our understanding of how to promote the well-being across a spectrum of outcome areas for youth (15 + years old) transitioning from out-of-home care to independent living through the study of the implementation and provision of (A) My Choice—My Way! (*Swe*. Mitt val–min väg!) and (B) Usual Services. This includes an increased understanding of how interventions for this population can be designed, implemented, and sustained in diverse settings.

The project has five specific aims and eleven research questions.

#### Aim 1

To test the short- and sustained effectiveness of My Choice-My Way! in normal practice settings.

H1: Youth participating in My Choice-My Way! will exhibit gains in well-being and psychosocial outcomes relative to youth participating in Usual Services.

*Research questions*. RQ1 What is the relative effectiveness of My Choice-My Way! and Usual Services (US) for youth aged 15 and older in out-of-home care on well-being and psychosocial outcomes?

RQ2 How are outcomes moderated by subgroup characteristics?

RQ3 How does implementation fidelity moderate outcomes?

RQ4 What is the relative economic impact of My Choice–My Way!?

#### Aim 2

To advance our understanding of how specific behavior change techniques and theory-based change mechanisms impact youth outcomes.

H2: Theoretically based change mechanisms (e.g., experiences of success targeting self-efficacy change) will have a mediating effect on outcome (e.g., increased engagement in educational activities) in interventions with detailed program content.

*Research questions*. RQ5 How are the specific behavior change techniques used by social workers related to theoretical change mechanisms?

RQ6 How do the specific change mechanisms in transition services mediate youth outcomes?

#### Aim 3

To assess the extent to which implementation support (i.e., GTO) can impact fidelity and sustainability in practice settings.

H3: Organizations engaging in implementation support will exhibit increased fidelity relative organizations not engaging in implementation support.
H4: Organizations engaging in implementation support will exhibit increased sustainability relative organizations not engaging in implementation support.

*Research questions*. RQ7 How does implementation support impact fidelity?

RQ8 How does implementation support impact sustainability?

#### Aim 4

To increase our knowledge of the barriers and facilitators to implementing programs for youth in diverse contexts.

H: The following research questions are exploratory and hypothesis generating in nature.

*Research questions*. RQ9 What strategies were used (e.g., by social workers) to implement transition services (e.g., My Choice–My Way!) and how do these strategies compare and contrast to current implementation models and frameworks?

RQ10 What are the experiences of providers that have implemented My Choice–My Way! without systematic implementation support (i.e. GTO)?

#### Aim 5

To build knowledge of how youth experience participating in interventions designed to support them in the transition from out-of-home care to independent living.

H: The following research questions are exploratory and hypothesis generating in nature.

*Research questions*. RQ11 What is the participants level of satisfaction, acceptability and buy-in with the service in which they have participated?

### Trial design

The study can be described as a hybrid effectiveness-implementation study with natural controls in a quasi-experimental two-arm study design [30]. We will conduct such a study to test the impact of the leaving care interventions while we at the same time collect data on the implementation process and outcomes (i.e., Hybrid I design). Research design and methods for data analysis specifically linked to each of the aims and research questions will be described in more detail below.

#### Design Aim 1

We will perform an effectiveness study to achieve Aim 1 and to answer RQ1-RQ4. The effectiveness study will be carried out as a natural quasi-experiment with two groups of youth participants (My Choice–My Way!, US) with dependent pre-, post- and follow-up measures [31] (i.e., 3 x 3 mixed factorial design. Long-term outcomes will be studied with a longitudinal register study.

#### Design Aim 2

Based on the My Choice–My Way! theory of change (i.e., logic model, [32]) salient change mechanisms will be chosen and measured.

#### Design Aim 3

Posttest comparison study comparing implementation and youth level outcomes for organizations engaged in implementation support with organizations not receiving implementation support.

#### Design Aim 4

Qualitative implementation, posttest interview study with service providers.

#### Design Aim 5

Qualitative implementation, posttest interview study with youth participants.

### Methods

This research project will be conducted in Sweden. The project has at its core the goal of informing practice about how work with implementation and intervention development can occur in order to benefit clients. In addition, we place a high value on keeping the work scientifically relevant and of high quality. Due to this, the project has an ambitious and complex data collection and analysis strategy using qualitative, quantitative and multiple informant methods.

### Study setting

This is a multicenter research project that will be conducted at a total of 21 research sites throughout Sweden. Organizations have naturally self-selected to either implement My Choice–My way! (*n* = 12) or to continue providing Usual Services (US, *n* = 9). All twelve organizations that implement My Choice—My Way! are part of the Swedish social services, although some are public and others private providers of services to youth. The nine organizations that constitute the control group and provide US are various organizations that provide support for the target group either within the Swedish social services or as a part of civil society. A list of study sites can be obtained from the first author of this protocol.

### Eligibility criteria

The project has two target groups: youth and service providers.

#### Youth participants

All youth in Sweden aged 15 years or older living in out-of-home care and placed by participating organizations, or who self-refer to the services provided by participating organizations, will be eligible for participation and invited to participate. Additional inclusion criteria for participating in My Choice–My Way!: the youth have the ability to spend periods of time outside of placement (i.e., institutional placement) without incident as determined by referring social worker. My Choice–My Way! should never be provided as a replacement for existing treatment services within the organization. Although our goal is to open inclusion to all youth aged 15 or older in out-of-home care, certain unforeseen characteristics may necessitate development of exclusion criteria (e.g., presence of severe intellectual disability).

#### Service providers

Those who are selected by their organizations to deliver My Choice–My Way! to youth participants are referred to as service providers. There were no specific inclusion criteria other than that the organization finds this person to be suitable for delivering the support and the service provider has received the authorized training in My Choice–My Way! The service providers included in the study had different roles within their organization. One thing all service providers have in common is that they interact with youth placed in different forms of out-of-home care in their daily work. All social workers charged with delivering My Choice–My Way! undergo a four-and-a-half-day training prior to starting their work with youth.

### Intervention

In the project, we will test and evaluate My Choice—My Way! (Swe. Mitt val-Min väg!) and Usual Services. Currently, there are no widespread, standardized transitions services for youth leaving care in Sweden.

#### My Choice—My Way!

My Choice–My Way! is delivered individually to youth aged 15 years and older by trained social workers. My Choice-My Way! is based on Self-Determination Theory [33], Social Cognitive Theory [34], and the COM-B Model for Behavior Change [18] as well as findings from prior research on the effects of interventions designed to support youth in their transition from out-of-home care to independent living [35–37]. The manual consists of guidance material provided to the program deliverer (i.e., service provider) as well as information and concrete materials (i.e., a workbook) provided to the youth participant [38, 39]. The youth material is optional, and use is based solely on the individual youths’ desire (i.e., autonomous decision-making) to use the material. The manual consists of 19 chapters divided into three blocks. The first block consists of sessions designed to orient the youth toward the future, the second block consists of a range of knowledge, skill and ability topics and exercises, and the third block consists of sessions designed to re-orient the youth toward the future as well as apply new knowledge, skills and abilities gained through participation in My Choice–My Way!. The 19 chapters are divided into core and optional content. Each chapter includes a short review of research motivating the topic area (as relates to youth transition from out-of-home care and youth developmental tasks), and concrete session goals.

Deliverers are tasked to work with youth through a minimum of 16 chapters (unless the deliverer determines that the youth participant already possesses the knowledge, skill, or ability covered in the material) over a period of 4–8 months. The sessions are designed to be delivered in an order that best suits the youth’s individual needs and deliverers are encouraged to plan sessions based on issues that are of most relevance to the youth in attaining their self-identified goals. My Choice–My Way! has four primary outcome areas: education, employment, healthy routines, and positive help-seeking behavior.

#### Usual services

Usual Services (US) comprise of a variety of services that are currently being provided for youth transitioning from out-of-home care within the Swedish social services or civil society. Interventions provided as part of US can be for example housing solutions in group homes or individual apartments with support from assigned staff members, or mentorship programs. Most Swedish Social services provide interventions with unspecified or undocumented content [40].

### Outcomes

#### Primary outcomes

All primary outcomes concern the youth participants.

**General Self-Efficacy Scale (GSE)** reflects an optimistic self-belief that one can perform a novel or difficult task, or cope with adversity [41]. Perceived self-efficacy eases goal-setting, effort investment, robustness in the face of barriers and recovery from setbacks. The scale consists of 10 items rated on a four-point sematic scale (“not at all true” to “exactly true”). A high score indicates high self-efficacy. The Swedish version was translated by Koskinen et al [42].

**Resilience Scale (RS-14)** reflects the degree of individual resilience, a positive personality characteristic that enhances adaptation [43, 44]. The scale consists of 14 items rated on a seven-point sematic scale (“disagree” to “agree”). A high score indicates high resilience. The Swedish version was translated by Lundman et al [45].

**The Need Satisfaction and Functional Scale (NSFS-18)** measures external motivation from three perspectives in three subscales: autonomy (the need that your own actions are self-initiated and self-regulated), social relatedness (the need in your own social environment to develop secure and satisfying connections), and competence (the need that you preform necessary actions with efficiency, [46]). The scale consists of 18 items rated on a five-point sematic scale (“agree” to “disagree”). A high score indicates high autonomy, social relatedness, or competence. The Swedish version was translated by Aurell et al [47].

#### Secondary outcomes

All secondary outcomes concern the youth participants.

**Daily Life Routines (DLR-18)** was inspired from the Sustainability of Living Inventory [48] but shortened and adjusted to fit youth’s everyday life. The total score of 18 items spans over areas of Personal Hygiene, Eating Habits, Sleeping Habits, Household Chores, Physical Activates, and Social Activities. A high score indicates robust routines. This version was developed by us.

**Social Support Questionnaire (SSQ-6)** maps the number of available others the respondent can turn to and the degree of satisfaction with the perceived support [49]. The number of support persons score assesses the number of available others the respondent feels he or she can turn to in times of need in each of six situations. The satisfaction score measures the respondent’s degree of satisfaction with the perceived support available in that particular situation. A high score indicates several support persons or high satisfaction. The Swedish version was translated by us.

**General Health Questionnaire 12** (GHQ– 12) is a screening instrument to detect psychosomatic symptoms and conditions [50]. The scale consists of 12 items phrased as statements about symptoms with four sematic response options on a four-point scale. Among the items, six are positively phrased and six are negatively phrased, but worded so there is no need to reverse scores. A high score indicates more psychosomatic symptoms. The Swedish version was purchased through https://mapi-trust.org.

**General Help Seeking Questionnaire (GHSQ)** assesses intentions to seek help from different sources including both formal and informal help source options [51]. The GHSQ uses a matrix format that can be modified according to purpose and needs to meet sample characteristics and study requirements. A high score indicates high intentions to seek help. The Swedish version was translated by us.

#### Other outcomes

*Youth self-reported measures*. ***Client Satisfaction Questionnaire (CSQ-8)*** assesses client satisfaction with services received by respondents [52]. The scale consists of eight items rated on a four-point sematic response option scale. A high score indicates high satisfaction. The Swedish version was purchased from https://csqscales.com.

*Youth level register data*. In order to assess longer-term outcomes, we will as part of this study collect specific variables from a number of official registers:

***LISA*** (Swedish acronym, *sv*. Longitudinell Integrationsdatabas för Sjukförsäkring- och Arbetsmarknadsstudier; *eng*. Longitudinal Integration Database for Health Insurance and Labor Market Studies) is a national registry database held by Statistics Sweden (https://www.scb.se/vara-tjanster/bestall-data-och-statistik/bestalla-mikrodata/vilka-mikrodata-finns/longitudinella-register/longitudinell-integrationsdatabas-for-sjukforsakrings—och-arbetsmarknadsstudier-lisa/) The database makes it possible to follow, over time, a person’s transitions between, for example, gainful employment, unemployment, sick leave and illnesses. Variables on education and training, employment and unemployment as well as income and social insurance can be used as outcomes.

***RAKS*** (Swedish acronym, *sv*. Registerbaserad AKtivitetsStatistik, *eng*. Register-based ACtivity Statistics) is a national registry database held by Statistics Sweden (https://www.scb.se/vara-tjanster/bestall-data-och-statistik/bestalla-mikrodata/vilka-mikrodata-finns/individregister/mikrodata-for-registerbaserad-aktivitetsstatistik-raks/). RAKS is a compilation and further development of variables that are already in the e.g., LISA database. The object is to describe an individual´s total livelihood and connection to the labor market. Two outcome variables of interest can be: establishment within the employment market and main source of income.

***Participation in Education*** (*sv*. Registerbaserad AKtivitetsStatistik) is a national registry database held by Statistics Sweden (https://www.scb.se/vara-tjanster/bestall-data-och-statistik/bestalla-mikrodata/vilka-mikrodata-finns/individregister/registret-over-befolkningens-studiedeltagande/). The statistics are based on registers for students in upper secondary school, adult education, qualified vocational education, labor market education and university and college. The information contained in this database enables the ability to describe the type of education, place of study, existence of study grants or loans, and educational provider for each individual within the study.

***Criminality*** is a combination between two ntional registry databases (*sv*. Belastningsregistret and *sv*. Misstankeregistret) held by the Swedish Police (https://polisen.se/lagar-och-regler/behandling-av-personuppgifter/polisens-register/). The registers contain information on whether a person has had criminal convictions, approved penalty orders and administrative fines, but also if they have been reasonably suspected of a crime or have been requested to be surrendered or extradited for a crime (with the exceptions of traffic violations). Only individuals over the age of 15 can be included in these registers.

***Hospital care*** derives from the national Patient registry (*sv*. Patientregistret) held by the National Board of Health and Welfare (https://www.socialstyrelsen.se/statistik-och-data/register/patientregistret/). The register contains both an individual´s outpatient and inpatient care. The registry contains variables such as duration of care, type of care, care disruption, diagnoses, and medication.

Register data will be collected 24 months after T1 for all youth who have consented to participate in the study, regardless of whether they have completed T2 and T3 or not.

*Service provider level implementation measures*. Service providers delivering *My Choice–My Way*! fill out the following implementation outcome measures post T2 based on Weiner et al. [53] on 5-point Likert scales: Acceptability of Intervention Measure (AIM, 4 items), Intervention Appropriateness Measure (IAM, 4 items), and Feasibility of Intervention Measure (FIM, 4 items).

Intervention dose estimation framework and tool assess intervention duration, percentage of intervention delivered, number of sessions (3 items).

#### Systematic session observation

Two sessions per youth will be randomly selected in the My Choice-My Way! arm of the study. The service provider will make a video recording of themselves (i.e., no video recordings of youth participants) providing the session to participant youth. The recordings will be sent to the research team for analysis of fidelity to model based on a structured assessment instrument.

### Participant timeline

**Fig 1:**
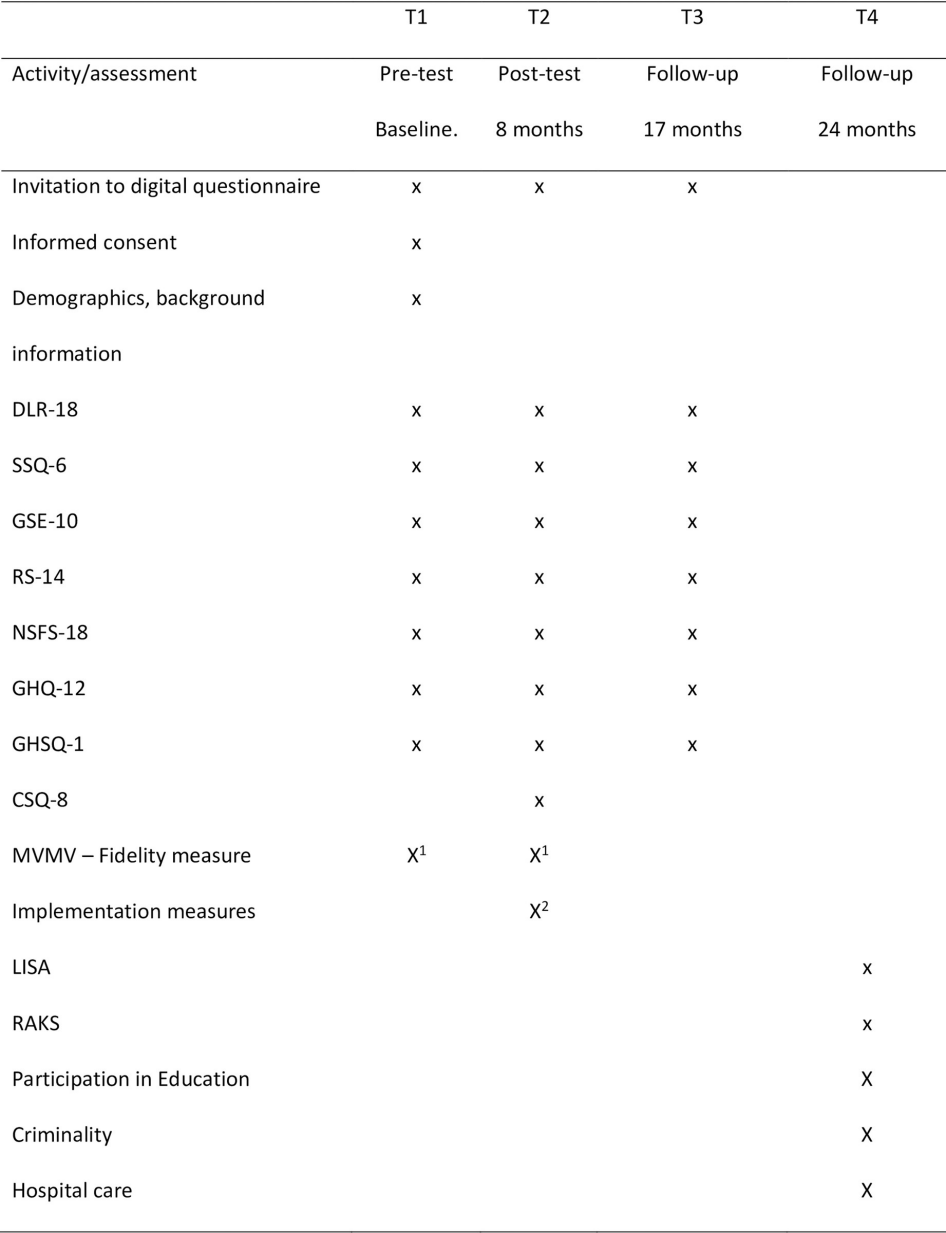
Schedule of enrollment, interventions, and assessments. 1. During the period between T1-T2. 2. PostT2.

The study will be conducted over the course of 24 months. Time schedule of enrolment, interventions, assessments, and visits for participants are presented in Fig 1. The start of the recruitment period for this study is October 2, 2022, and the prospective end of the recruitment period for this study is December 31, 2023.

### Sample size

Following Cohen’s proposed interpretation of *f*, this study will need a minimum of 124 youth to fully participate (complete) in the effectiveness trial in order to test within and between interactions over three measurement points [54, 55]. Assuming a 20% drop out rate, we aim to recruit 80 youth per study arm. This will result in 85% statistical power to detect a medium (*f* > .25) effect with alpha set at .05 for a repeated measures ANOVA with within and between interactions.

Service provider sample size is based on the ratio of service provider to youth participant referred to the study.

### Recruitment

Youth are recruited directly by participating youth service organizations. Organizations are self-referred to receive training in My Choice–My Way!. This is following a wide scale recruitment effort conducted via announcements on social media, direct contact to national, regional, and local youth serving and policy organizations. Youth are informed about the study verbally and in writing and subsequently asked if they are interested in participating by staff at the participating organizations (i.e., service providers). Participating organizations are responsible for recruiting service providers that are deemed suitable to work with delivering the intervention.

### Allocation

This study will follow the natural assignment of youth to condition (i.e., the research team will not manipulate the normal operating procedures of the participating municipalities/organizations but instead will follow youth as they are assigned to My Choice–My Way! and US). We will implement a rolling inclusion. Youth will be admitted to the study as they are referred to one of the study arms (My Choice–My Way!, US). Inclusion to the study will continue until we have included the desired number of participants.

### Data collection methods

Youth level data will be collected through a digital questionnaire at three (i.e., T1, T2, T3) time points. First, pre-test measurements (T1), directly following the informed consent procedure but prior to program start. Post-test measurement (T2), after completion of My Choice–My Way! (approximately 8 months after T1, standardized across groups). Third, follow-up measurements (T3), 9 months following T2. Youth level register data will be collected 24 months after T1.

Upon declaration of interest to participate in the study, the youth will be given the option to give their informed consent and fill in the baseline questionnaire electronically via a link received through e-mail or over the phone with a member of the research team. Each youth who chooses to participate will receive compensation for their time upon filling out each questionnaire (approximately $20). Participants are able to choose between receiving compensation in cash or in the form of a gift card.

Service providers of My Choice—My Way! will be asked to complete a questionnaire post-T2 data collection investigating key determinants of implementation success.

### Statistical methods

#### Aim 1 statistical methods

*Baseline characteristics*. Demographic/background and T1 values on outcome variables will be investigated for the entire group and each arm separately.

*Baseline differences in between-group analyses*. Baseline differences on background and outcome variables between groups at T1 (due to the absence of randomization) will be investigated (e.g., *x2*, *t*-test, Mann-Whitney U). Any identified differences will be statistically controlled for in between group analyses.

*Attrition*. Analysis of within group and between group attrition will be conducted (i.e., differences in baseline characteristics of those that remain in study and those that drop out e.g., *x2*, *t*-test, Mann-Whitney U).

*Intent-to-treat (ITT)*. All analyses will be conducted with an ITT approach and missing values on follow-up data will be imputed using the maximum likelihood (ML) approach.

*Sensitivity analysis*. All results will be investigated through sensitivity analysis for their robustness to assumptions and method choices made during the course of the study (e.g., results with imputation vs results without imputation, changes in resource use and cost data).

*RQ1*. The relative effectiveness of My Choice–My Way! and US will be assessed by analyzing within group and between-group changes on participant self-reported and register data after T2 and again after T3 data collection using e.g., ANOVA, ANCOVA depending on final data characteristics.

*RQ2*. Participant characteristics measured at T1 (e.g., gender, age, type of placement) to will be analyzed (e.g., regression-based moderation analysis, ANCOVA depending on final data characteristics) for their moderating effect on changes in self-reported and register data collected at T2 and T3.

*RQ3*. Service provider reported implementation measures (i.e., acceptability, appropriateness, feasibility) and fidelity scores from the systematic session observations will be analyzed (e.g., regression-based moderation analysis, ANCOVA depending on final data characteristics) for their moderating effect on changes in participant self-reported and register data collected at T2 and T3.

*RQ4*. Cost analysis will include the resources necessary to provide the intervention along with a comparison of the relative cost-effectiveness of the intervention compared to the control condition. The incremental costs of providing the intervention will be assessed prospectively considering incremental changes in youth outcomes through economic analysis (e.g., cost-benefit, cost-effectiveness, or cost-consequence depending on final outcome).

#### Aim 2 statistical methods

*RQ5 and RQ6*. Structural equation modeling (SEM) will be used to investigate the structural relationship between the measured behavior change techniques, latent behavior change mechanisms and youth outcomes.

#### Aim 3 statistical methods

*RQ7*. Fidelity ratings from the systematic session observation at T2 will be compared across My Choice–My Way! groups to investigate differences in implementation quality (e.g., independent samples t-test, Mann-Whitney U depending on final data characteristics).

*RQ8*. Fidelity measures collected from My Choice–My Way! T3 will be compared (e.g., ANOVA) for significant differences between groups.

#### Aim 4 statistical methods

*RQ9 and RQ10*. Service provider interview data will be analyzed with a thematic analysis approach as described by Braun and Clarke [56].

#### Aim 5 statistical methods

*RQ11*. Client satisfaction data collected from youth during T2 data collection will be analyzed with descriptive statistics (e.g., % of participant youth with a given opinion of the support they received). Youth interview data will be used to contextualize and gain further understanding of the perspectives captured in the client satisfaction survey via thematic analysis.

## Discussion

Supporting vulnerable youth in their healthy development is a complex endeavor. This project integrates several scientific fields including social work, developmental psychology, and implementation and intervention science and merges these scientific fields with practitioner expertise to meet this challenge. This research project provides the opportunity to increase scholarship around how we might design interventions for this population specifically and implement services in practice settings specifically but also increase our general scientific knowledge across a range of areas important for the scientific development of intervention and implementation science by attending to a range of areas for which the current scholarship is lacking (e.g., strong research design, mediation and moderation analyses). In addition, this research has an ambitious data collection and analysis strategy which incorporates, qualitative, quantitative and observational data across intervention and implementation in order to support analyses that are exploratory, descriptive and explanatory in nature thereby increasing our ability to study multiple processes and their interaction within the same trial. Importantly, the proposed research project is part of a larger programmatic line of basic and applied scientific work being conducting by our research team with the aim to advance the well-being and psychosocial outcomes of youth transitioning from out-of-home care to independent living.

A main goal of the current research program is to provide social service agencies in Sweden with an effective and feasible intervention that targets youth transitioning between out-of-home care and independent living that can be sustainable over time. To date, such interventions are lacking in the scientific literature. Thus, the research project has in its design to produce knowledge and tools, related to both methods and sustainable implementation, which can be of direct practical use in society. In fact, sustainable use of the intervention in regular services is one of our research questions. We bridge the gap between research and practice via close collaboration with practitioners from social service organizations throughout the project period. The intervention and the implementation strategies will be developed together with the practitioners and will be informed by what matters in “the real world”. Thus, the results of the study will be very useful for the social services in all municipalities in Sweden.

My Choice–My Way! Is the result of an extensive development process which included attention to culture in the early development phase. Cultural fit is key to intervention success [57, 58] but there remains debate in the extant literature on whether this fit should be managed through the cultural adaptation of existing interventions to new context or whether fidelity should be seen as paramount [59]. In addition, a large proportion of interventions are developed in the settings in which they will be used [59, 60]. The current research may be able to contribute to this scholarship in that we test the intervention My Choice–My Way! in the context in which it was developed as well as implement it more widely across social service settings in the same trial.

Our development process was guided by a partnership approach which is described as a collaboration in which researchers and stakeholders work together on a research project with the aim of creating collaborations in which researchers and stakeholders co-lead research activities and collectively apply their expertise, knowledge, and skills within the team [61]. Through this approach we developed an intervention that weaves together scientific theory, implementation best practices and insights from evidence-based practices [62] and as such we believe that the resulting intervention My Choice–My Way! with its theoretical basis in self-determination theory [33], Social Cognitive Theory [34], and the COM-B Model for Behavior Change [18], is highly interesting and promising given that Swedish youth placed in out-of-home care view themselves as not possessing the essential resources and capabilities required to tackle the challenges that arise during the transition from such care [57].

### Gender and diversity perspectives

Even if this project does not directly address gender or diversity as core issues, such issues are essential to intervention and implementation research. In terms of gender, it has been shown that, although more boys are placed in out-of-home care, girls placed in out-of-home care in Sweden are a more adverse and vulnerable group than boys. Important gender differences will be consistently woven into this project and subgroup analyses will include attention to gender. Reporting of results and dissemination will highlight any identified gender issues. Previous research has made clear that the cumulative effects of disadvantage for adolescents in out-of-home care may impact girls and boys differently and the mechanism through which this disadvantage operates appears to be through school achievement. Additionally, girls and boys may accept intervention unequally and access/participate in intervention components unequally. Therefore, this project will keep a strong focus on sex and gender differences and subgroup analyses will be performed.

## Supporting information

S1 Checklist SPIRIT checklist. (DOC)

S1 File Study protocol approved by ethics committee. (DOCX)

S2 File Swedish translation of the original ethics approval. (DOCX)
