# Supplementary material for: Study protocol for leaving care—A comparison study of implementation, change mechanisms and effectiveness of transition services for youth
Source: PLoS One. 2024 Feb 8;19(2):e0293952. doi: 10.1371/journal.pone.0293952 (PMC10852220; doi:10.1371/journal.pone.0293952)
Supplement: S1 File — (DOCX) [file pone.0293952.s002.docx]

The proposed project is directly related to the following prioritized areas of research contained in Forte’s *Strategic agenda for the national program on applied welfare research*:

- Effectiveness of interventions.
- Implementation, change, and improvement strategies.
- Non-governmental organizations (NGOs) as providers of social service interventions

In addition, the proposed project is directly related to three of the top ten priorities listed in Forte and SBU’s joint publication, “Prioriteringar för forskning om socialtjänsten – Perspektiv från brukare, policy och praktik”:

1. How can the municipal social services (i.e., socialtjänsten) work with (a) implementation, (b) elimination and (c) introduction of evidence-based knowledge?
2. What effects do current and new interventions have for meeting client needs?
3. What are the effects of NGO driven welfare?

We address priority (1) above by focusing specifically on item (1a) and (1c) in RQ3 and we address priority (2) and (3) above via RQ1, RQ2, RQ4, RQ5, RQ6 as described in the following sections.

This project has both a client and practice focus and is a continuation of prior successful projects/studies, which have been conducted in close collaboration with clients (youth) and professionals. Collaboration with youth has been in adjusting and choosing outcome measurements and designing intervention program content based on their perspective. In the current project, our team has the goal of including youth as not only participants in the research project but also through participation in a youth advisory committee. Over the past year our team has been collaborating closely with the municipal social services in Farsta, Stockholm. Our work has been focused specifically on co-creating an intervention for young people transitioning from societal care to independent living. This group consists of researchers, municipal social workers, municipal section heads and municipal organizational developers. An important goal of the working group has been to give research, practice, and organizational perspectives *equal importance* in the development process. The project proposed in this application will include the NGO SOS Children’s Villages who provide transition services to youth in both Stockholm and Göteborg due to the lack of support for this vulnerable group. As such this project is client focused, municipal actor (social worker, sections heads, etc) focused, and NGO-actor focused.

We have been actively working on this project since 2018 and the activities proposed here are a continuation of activities contained within a larger research program.

**Purpose of this study:** Increase our understanding of how to promote the well-being across a spectrum of outcome areas of youth (15 + years old) transitioning from societal care to independent living through the provision of two supportive intervention programs (1) *Mitt val – min väg*! and (2) transition services provided by SoS Childrens Villages. Including an increased understanding of how interventions for this population can be designed, implemented, and sustained in municipal social service and NGO settings.

Youth placed in societal care is a large and highly vulnerable group with high risks of negative, developmental outcomes. About 3-4% of Swedish children are placed in societal care (i.e. care situations where youth live outside of their own home e.g. foster care, group home care, institutional care) at some point and about 1% of children grow-up primarily in societal care (1). Most of the children placed in societal care are teenagers. Swedish research continually shows that these youth transition to independent living and adulthood with relative disadvantage in all types of health related outcomes (e.g. somatic, mental, and dental; see meticulous review by 2), educational attainment (3), housing stability (4), early childbearing and reproductive health (5), substance abuse (6), offending (7), exclusion from or weak attachment to the labor market (8), and public welfare dependency (7) compared to their non-placed peers. This negative development pattern is consistent across studies regardless of outcome, choice of comparison group, methodological approach, or location of study.

Given the size and extent of negative developmental outcomes for youth placed in societal care, interventions to help this vulnerable group navigate successfully towards independent living and promote wellbeing across a spectrum of outcome areas are needed. Unlike other countries there is no legislation regarding the provision of transition services for this group in Sweden. Current practice places emphasis on activities which occur prior to placement (e.g., recruitment of foster families) with little to no interventions provided for support of children during ongoing care (9). None of the strategies for care of children in societal care currently in use in Sweden have been evaluated for their effectiveness (ibid). There is very little research regarding the effectiveness of interventions for transition aged youth. A review (10) concluded that youth aging out of societal care who participate in the programs investigated obtain some productive outcomes but that comparisons with non-participant youth and comparisons between interventions could not be made because the studies included in the review were poorly designed. Another review of interventions designed to improve the physical and mental health of children placed in foster care (9), found that outcomes in this group can be improved but only three of 18 specific interventions had more than “some reliable evidence of effectiveness” (p. 7). Only one of these targeted youth themselves. This result was mirrored in a review of transition services for youth leaving societal care which found that Independent Living Services which adapt services to youth needs can have positive results (11).

Across systematic reviews (9, 11, 12) the potential of *self-determination* interventions to improve outcomes for this population has been highlighted. Self-determination originates from multiple youth-oriented fields (e.g., 13) where consensus has emerged around self-determination as a developmental protective factor (14). A growing body of research has affirmed the promotive role of self-determination in positive youth development (e.g., 13), and in quality-of-life outcomes (including internalizing and externalizing behaviors) for youth (15). Intervention to enhance self-determination focuses on the intention to make decisions, to direct one's actions, and to exercise rights and responsibilities, within the context of an individual’s culture, experiences, and aspirations. Experimental tests of interventions based on self-determination theory are few, have small sample sizes, and have not been replicated. Two intervention components have been replicated across self-determination enhancement studies (1) youth-directed skill-building to develop specific competencies for accomplishing transition-related goals (selecting goals, problem-solving, self-regulation; 16), and (2) consistency in promoting positive youth attitudes and beliefs around their capacity for self-determination in their lives. Specific tests of mechanism-based moderation are lacking. This means that even though interventions of this type appear to be promising and have some empirical support for their effectiveness, the causal link between specific program components (e.g., goal setting), theoretical constructs (e.g., behavior regulation) and outcome (e.g., educational achievement) has yet to be tested. Increasing our understanding of *how* specific activities impact change processes has the potential of aiding our ability to design effective programs in the future.

Theory development and interventions for youth: Linking behavior change techniques (professional practice components) with mechanisms of action (theoretical constructs) and outcome (client level behavior change). Despite literature describing the steps involved in developing interventions (e.g., 17, 18) little is known about how we might put into practice specific activities (i.e., program content) to impact specific change mechanisms (i.e., constructs) in order to impact outcomes for individuals, groups and society. There exists an abundance of behavior change techniques (e.g., goal setting, problem solving; 18, 19) available as well as theoretical models of behavior and behavior change (e.g., self-determination theory, social cognitive theory; 20) but empirical testing of the links between these two is lacking (19). Increased understanding of these links is key to informing the development of effective interventions by providing information on the strategies and target mechanisms likely to be effective in specific contexts and among specific populations (21). A similar shortcoming concerns the link between change mechanisms and outcome. Recent meta-reviews of interventions across a range of contexts and populations have brought to light the limited extent to which primary studies (1) include theory-based measures of the dependent variable, (2) tend not to conduct the appropriate analyses to test intervention change mechanisms, (3) do not provide enough information for secondary analyses of change mechanisms and (4) do not specify a proposed theory-based mechanism for the effect of the intervention on individual behavior (22-24). Although primary studies of the effectiveness of interventions test the effects of intervention on outcome, assessment of *how* a successful intervention changes behavior is lacking (i.e., via a priori planned and appropriately powered mediation analysis; 23).

In order for effective interventions to produce positive change in individuals, the implementation process within human service organizations (e.g., municipal social services, NGOs) must also be effective. Few programs survive after the initial implementation period to become a sustained, integral part of regular service (for review, see 25). Organizations could cease the use of the new program entirely or they could significantly alter it in a way that may or may not be desirable given the current context and target group (for a Swedish example see, 26). The understanding that interventions can fail due to theoretical deficiencies in the intervention itself or shortcomings in the implementation process has led to increased focus on *how* we might systematically carry out implementation efforts in order to improve implementation effectiveness when moving interventions into practice (for a review of implementation theories, models and frameworks see 27). Getting to Outcomes (GTO, 28) is both an implementation model for carrying out intervention activities, and a support intervention aimed at enhancing practitioner capacity. Built on social cognitive theories of behavioral change and implementation science theories such as the Consolidated Framework for Implementation Research (29), developers purport that GTO builds practitioner capacity to perform multiple implementation best practices needed for interventions. Improved performance of these implementation best practices when delivering a specific intervention can improve intervention fidelity, which results in more positive outcomes for clients. GTO has been applied to several content areas such as drug and alcohol prevention programs, teen pregnancy prevention, prevention of underage drinking, and positive youth development. GTO has been found to positively impact implementation and sustainability of programs at the organizational level (e.g., 30). As part of the first GTO study, a quasi-experimental trial from 2002 to 2005, face-to-face training and ongoing technical assistance to the existing written guide was added. These components have been employed in subsequent tests of GTO. In these studies, GTO developers have been actively involved, leaving questions as to whether GTO as a general implementation support (i.e., without developer support or involvement) can aid in boosting program effectiveness and sustainability.

Our past work forms a solid foundation for the new, proposed project. This project builds solidly on three prior studies/projects. The first was financed by Stiftelsen Allmänna Barnhuset in 2018. The catalyst for this study was a systematic review on interventions to improve the physical and mental health of foster children which highlighted three interventions, one of which targeted youth themselves (9). This intervention is called *Take Charge* and is a self-determination model for supporting youth in the transition from societal care to independent living developed and tested in the USA.

Our study (33) attempted to assess whether the intervention *Take Charge* could be interesting in the Swedish context. Results indicated that US and Swedish youth in societal care differ in many important aspects: (1) Swedish youth experience significantly fewer (concrete) barriers to education compared to US youth. Similarly, Swedish youth have performed significantly more (concrete) independent living activities when compared to US youth; (2) Swedish youth are significantly more negative toward school/education and their school environment compared to US youth; (3) compared to US youth, Swedish youth score significantly lower on measures of empowerment, self- efficacy, and resilience. These results are especially interesting in light of Swedish research that finds educational achievement to be one of the main protective factors for future adjustment in this population (5, 31). Along with cost considerations and ongoing license requirements with US developers, these results raised questions as to the feasibility and expected outcomes of *Take Charge* in the Swedish context.

The second project was financed by a VINNOVA *Verifiering för samverkan* grant in 2019. We were granted funds to initiate our collaboration with Farsta. The group’s activities followed the steps in the GTO framework which detailed the specific activities that the working group engaged as well as the specific rolls and responsibility areas within the working group. Specific activities undertaken as part of the project included: creating a shared vision for the project, identifying resources and needs, developing a plan for future coordinated work, developing concreate project goals as well as developing an ongoing financial plan.

The third project was a continuation of GTO activities and was financed by a Forte planning grant in 2020. The purpose of this project was to finalize the initial development of an intervention for youth transitioning from societal care and pilot test the intervention with a small group of youth placed in societal care and staff implementing the intervention in Farsta in order to (1) make necessary adjustments to the design and implementation of the intervention and (2) prepare for a larger effectiveness study of the intervention. This systematic, collaborative process resulted in the first full draft of a manual-based intervention called, *Mitt val – min väg!* (MVMV) which is based on:

1. The outcomes defined as important for the population by our collaborative partners in Farsta (e.g., help seeking behavior, developing routines, education, employment).
2. Basic research on risk and protective factors for the outcome areas identified by our collaborative partners.
3. Descriptions and program theories of interventions found in meta-analyses to be effective in strengthening the identified protective factors in (2) above and moving youth toward identified outcome areas in (1) above; and
4. Behavior change theories directly related to the change mechanisms identified in (3) above (i.e., self-determination theory, social cognitive theory, and the Competence-Opportunity-Motivation-Behavior system).

See Figure 1 Logic Model, MVMV and Table 1 Example of specific content, MVMV

In September 2020, our research team was approached by the Swedish arm of the NGO SOS Children’s Villages (SOS). SOS currently provides services to this population in both Stockholm and Göteborg. Their intervention is fully implemented and in operation (see Figure 2 Logic Model, SOS). At that point, SOS was included in our pilot project. Our goal is that this collaboration will help us learn more about not only the effectiveness of competing interventions but also, how services can be organized across service delivery systems for this population.

This project is novel in a number of ways. First, this project weaves together several complementary areas of research to meet the needs of the municipal social services and the youth in its care (i.e., implementation science, intervention science, developmental psychology). Second, this research will experimentally test the effects of a locally developed, theoretically and empirically inspired intervention MVMV developed based on cutting-edge intervention development theory in collaboration with municipal stakeholders aiming to support an underserved and extremely vulnerable group, one of the municipal social services core populations. Third, this research will simultaneously experimentally test the effects of a similar intervention program independently developed by a NGO. Fourth, this project will experimentally test the extent to which implementation support impacts fidelity, sustainability and client outcomes. Fifth, this research will increase our empirical understanding of how specific behavior change techniques (practice components) are related to theoretical change mechanisms (constructs) and how these constructs in turn are related to youth outcomes. This has the prospect of increasing our scientific knowledge of how intervention development and implementation can be approached in the future. Finally, there are currently no evidence-based and culturally sensitive interventions available to social service professionals for this population and purpose. Our work has the goal of developing our knowledge toward meeting this challenge.

Aim 1: Test the short- and sustained effectiveness of MVMV in normal practice settings.

H1: Youth participating in MVMV will exhibit gains in well-being and psychosocial outcomes relative youth participating in Usual Services or transition services provided by SoS.

Research questions:

- (RQ1) What is the relative effectiveness of MVMV, SOS and Usual Services (US) for youth aged 15 and older in societal care on well-being and psychosocial outcomes?
- (RQ2) How are outcomes moderated by subgroup characteristics?
- (RQ3) How does implementation fidelity moderate outcomes?
- (RQ4) What is the relative economic impact of MVMV, SOS, and US?

Aim 2: To advance our understanding of how specific behavior change techniques and theory-based change mechanisms impact youth outcomes.

H2: Theoretically based change mechanisms (e.g., experiences of success targeting self-efficacy change) will have a mediating effect on outcome (e.g., increased engagement in educational activities) in interventions with detailed program content.

Research questions:

- (RQ5) How are the specific behavior change techniques used by social workers related to theoretical change mechanisms?
- (RQ6) How do the specific change mechanisms in MVMV and SOS mediate youth outcomes?
- (RQ11) What is the participants level of satisfaction, acceptability and buy-in with the service in which they have participated?

**Studiedesign, metoder för datainsamling och analys**

Research design. This project has uses two primary designs. First, participating municipalities will be cluster randomized to provide either MVMV or US. Second, MVMV provided in Farsta stadsdel and participants in SOS will be matched with participants from US in a natural (quasi) experiment.

Target population. All youth aged 15 years or older living in societal care and placed by participating municipalities or youth that self-refer to SOS will be eligible for participation and invited to participate. Although our goal is to open inclusion to all youth aged 15 or older in out-of-home care, certain unforeseen characteristics may necessitate development of exclusion criteria (e.g., presence of severe intellectual disability).

Assignment. Youth will be assigned to condition (i.e., MVMV, SOS and US) based on the municipality from which they are refered and based on the intervention (MVMV, SOS or US) that that municipality is providing.

Inclusion into study. We will implement a rolling inclusion. Youth will be admitted to the study as they are referred to/self-refer one of the study arms. Inclusion into the study will continue until we have included the desired number of participants (see power calculation below).

Measurement. Measurement will occur at three (i.e., T1, T2, T3) time periods. First, pre-test measurements (T1) will occur directly following the informed consent procedure but prior to program start. Post-test measurement (T2) will occur after completion of MVMV (approximately 8 months after T1, standardized across groups) and (T3) will occur 9 months following T2.

Groups. Comparison will be made between youth participating in MVMV_,_ SOS and US. Subgroup analysis will be conducted.

Moderation. Subgroup analysis will be conducted to investigate the extent to which individual characteristics (e.g., gender, age, placement type) and programmatic characteristics (e.g., participation intensity, implementation quality) moderate outcomes.

Mediation. Based on MVMV and SOS theories of change (i.e., logic model) salient change mechanisms will be chosen and measured (see participant measures below).

Participant information and measures at pre-test (T1)

*Personal information*: name, personal number, municipality of residence, placing municipality, contact information.

*Background/demographic variables*: Gender, country of birth, parental country of birth, parental residency at T1, date of first placement, number of placement changes, date of current placement, current grade level, type of placement (foster care kinship, foster care non-kinship, HVB, institution), first language, immigration status, school achievement, receipt of social- and/or pedagogical intervention, placement changes (i.e., breakdown in care).

*Participant self-reported measures at pre-test, post-test, and follow-up (T1, T2, T3).*

- *Self-efficacy,* General Self-Efficacy Scale (GES-10)
- *Resilience and hope*, e.g., Resilience Scale (RS-14)
- *General health,* e.g., General Health Questionnaire (GHQ-12).
- *School achievement* will be assessed via youth self-reports e.g., “Do you currently attend school”; “What is your current grade level?”; “How often do you skip class?”.
- *Social support*, e.g., Social Support Questionaire (SSQ-6)
- *Life satisfaction,* e.g., Need Satisfaction and Frustration Scale
- *Help-seeking behavior*, e.g., General Help-Seeking Questionnaire (GHSQ).
- *Client Satisfaction*, e.g., Client Satisfaction Questionnaire (CSQ-8, post-test, T2, only).

*Participant level register data.* In order to assess longer-term outcomes, we will as part of this study collect specific variables from a number of official registers:

- Longitudinal integrated database for health insurance (LISA, Statistics Sweden). Variables on education and training, employment and unemployment as well as income and social insurance.
- Microdata for register-based activity statistics (RAKS, Statistics Sweden). Two main variables are of interest: establishment within the employment market and main source of income.
- Register of the populations’ participation in education (Statistics Sweden). Variables on type of educational participation, receipt of educational grants, and classification of type of education.
- Crime registers (Swedish Police). Variables on charges and sentences.
- Patient register (National Board of Health and Welfare). Variables on outpatient and inpatient care (e.g., substance use/abuse, mental health, reproductive health)

*Sample size calculation*. In order for any intervention to be interesting to practice, effect sizes have to be large enough to warrant widespread implementation. Following Cohen (32) proposed interpretation of *f*, this study will need a minimum of 196 youth to fully participate (complete) in the effectiveness trial in order to test main effects. Assuming a 20% drop out rate, this study would need to recruit 245 (82 per group) youth. This would result in 80% statistical power to detect a medium (*f* >.25) effect with alpha set at .05 for a fixed effects ANOVA with within-between interactions. Our initial work has indicated that (generally speaking) municipalities with 40.000 residents or more have well over this number of eligible youth in care at any point in time (e.g., 33) and initial numbers from our collaborative partners confirm this. Our challenge is seeing to it that (1) participating organizations have sufficient manpower to provide interventions to youth over the inclusion period, (2) our inclusion period is long enough to be able to manage participant flow and not overwhelm professionals, and (3) we keep drop-out to a minimum.

*Participant reported implementation measures*. Implementation measures will be used as covariates in order to (1) assess the extent to which MVMV and SOS were implemented as intended, (2) assess the extent to which these components are present in US, and (3) assess the extent to which identified implementation components impact outcome.

Service provider (i.e., social worker, mentor) and organizational measures

*Service provider reported implementation measures.* Service providers of MVMV and SOS will be asked to complete a questionnaire post-T2 data collection investigating key determinants of implementation success over a range of programs and includes items from:

- Acceptability of Intervention Measure (AIM)
- Intervention Appropriateness Measure (IAM)
- Feasibility of Intervention Measure (FIM)
- Intervention dose estimation framework and tool

*Resource use and costs*. Data will be collected regarding the implementation and running costs of MVMV, SOS, and US (e.g., time, material, and other inputs) from participating organizations between T1 and T2.

*Systematic Session Observation.* Two sessions per youth will be randomly selected in MVMV and SOS arms of the study. The service provider will take a video recording of themselves (i.e., no video recordings of youth participants) providing the session and these recordings will be sent to the research team for analysis of fidelity to model and behavior change techniques used.

Quantitative analysis

*Baseline characteristics.* Demographic/background and T1 values on outcome variables will be investigated for the entire group and each arm separately.

*Baseline differences in between-group analyses*. Baseline differences on background and outcome variables between groups at T1 (due to the absence of randomization and the participant level) will be investigated (e.g., *x*^2^, *t*-test, Mann-Whitney U). Any identified differences will be statistically controlled for in between group analyses.

*Attrition.* Analysis of within group and between group attrition will be conducted (i.e., differences in baseline characteristics of those that remain in study and those that drop out e.g., *x*^2^, *t*-test, Mann-Whitney U)

*Intent-to-treat (ITT)*. All analyses will be conducted with an ITT approach and missing values on follow-up data will be imputed using the maximum likelihood (ML) approach.

*Sensitivity Analysis.* All results will be investigated through sensitivity analysis for their robustness to assumptions and method choices made during the course of the study (e.g., results with imputation vs results without imputation, changes in resource use and cost data).

*RQ1 and RQ7*: The relative effectiveness of MVMV, SOS, and US will be assessed by analyzing within-group and between-group changes on participant self-reported and register data after T2 and again after T3 data collection using e.g., ANOVA, ANCOVA depending on final data characteristics.

*RQ2:* Participant characteristics measured at T1 (e.g., gender, age, type of placement) to will be analyzed (e.g., regression-based moderation analysis, ANCOVA depending on final data characteristics) for their moderating effect on changes in self-reported and register data collected at T2 and T3.

*RQ3*: Participant reported implementation measures and fidelity scores from the systematic session observations will analyzed (e.g., regression-based moderation analysis, ANCOVA depending on final data characteristics) for their moderating effect on changes in participant self-reported and register data collected at T2 and T3.

*RQ4:* Cost analysis will include the resources necessary to provide the intervention along with a comparison of the relative cost-effectiveness of the intervention compared to the control condition. The incremental costs of providing the intervention will be assessed prospectively considering incremental changes in youth outcomes through economic analysis (e.g., cost-benefit, cost-effectiveness, or cost-consequence depending on final outcome).

*RQ5 and RQ6:* Structural equation modeling (SEM) will be used to investigate the structural relationship between the measured behavior change techniques, latent behavior change mechanisms and youth outcomes.

**Arbetsplan**

This project has at its core a goal of informing practice about how work with implementation and intervention development can occur in order to benefit clients. In addition, we place a high value on keeping the work scientifically relevant and of high quality. Due to this, the project has an ambitious and complex data collection and analysis strategy. The work plan is presented in more detail below. In parentheses next to each task, we have marked with initials (TO Tina Olsson; MB Martin Bergström; TS Therese Skoog; Matilda Karlsson PhD Candidate) who will be involved in which activities.

Year 1 (3-9 months): Preparatory activities include application for ethics board approval. Training of social workers for MVMV. Participant (youth) recruitment materials, agreements and control documents from pilot revised as needed. Translate and validate measures as necessary. Write and publish study protocol for international publication (TO, MB, TS).

Year 1: Advisory groups meet (separately). (TO, MB, TS)

Year 1 (12 months): Ongoing activities include ongoing implementation support (GTO) activities in Farsta and coalition building activities (across organizations). (TO, MB, TS)

Year 2 (12 months): Rolling recruitment and inclusion of youth in MVMV, SOS, US. T_1_ data collection begins and continues. (MK has primary responsibility with support from TO, MB, TS as needed).

Year 2 (12 months): Ongoing activities continue include ongoing implementation support (GTO) activities in Farsta and coalition building activities (across organizations). (TO, MB, TS).

Year 2 (4 months): T_2_ data collections begins and continues. (MK has primary responsibility with support from TO, MB, TS as needed).

Year 2: Advisory groups meet (separately). (TO, MB, TS) (MK as time allows). Publication strategy developed (TO, MB, TS).

Year 2-3 (24 months): Ongoing collection of recorded data (systematic session observation) between T_1_ and T_2_. Service provider reported implementation measures collected as youth pass T_2_ data collection (TO, MB, TS, MK).

Year 3 (4 months): Rolling recruitment and inclusion of youth (T_1_) in MVMV, SOS, US and T_1_ data collection continues and concludes (MK has primary responsibility with support from TO, MB, TS as needed).

Year 3 (12 months): T_2_ data collections continues and concludes (MK has primary responsibility with support from TO, MB, TS as needed).

Year 3 (5 months): T_3_ data collection begins and continues (MK has primary responsibility with support from TO, MB, TS as needed).

Year 3 (2 months): Collection of resource use data for Year 2 program costs for the economic analysis (TO, MK).

Year 3: Advisory groups meet (separately). (TO, MB, TS) (MK as time allows).

Year 4 (6 months): Data analysis T_1_ – T_2_ (short-term outcomes) and peer-reviewed publications written (TO, MB, TS). (MK as time and interest allows).

Year 4 (9 months): T_3_ data analysis continues and concludes. (MK has primary responsibility with support from TO, MB, TS as needed).

Year 4 (4 months): Data analysis T_2_ – T_3_ (long-term outcomes) and peer-reviewed publications written. (TO, MB, TS). (MK as time and interest allows).

Year 4 (2 months): Collection of resource use data for Year 3 program costs for the economic analysis. Economic evaluation completed on short- and long-term results. (TO, MK).

Year 4 (3 months): Collection and analysis of register data aver T_1_ – T_2_. (TO, MB, TS).

Ongoing activities: Communication results – articles and conference presentations - to both practitioners and the science community. (TO, MB, TS)

Post research project activities: The proposed research project is part of a larger programmatic line of basic and applied scientific work conducting by our research team with the aim to advance the well-being and psychosocial outcomes of youth transitioning from out-of-home care to independent living. Ongoing post project activities include communication results to both practitioners and the science community, further development manual based on what has been learnt during the project. Post project activities also include issues of sustainability and scale-up depending on results.

Project organization: In the current proposed project, TO will have overarching responsibility for project administration (both internally and externally), coalition building activities, working group facilitation and maintaining GTO structure. TO will facilitate the scientific advisory board activities and TS will facilitate the youth advisory board. TO, MB and TS will participate in all working group/coalition/advisory group meetings. MB will have overarching responsibility for identification, obtainment, translation and testing of measurement instruments to be used in the implementation and effectiveness study as needed. TS will have primary responsibility for MVMV training sessions. MK will provide ongoing support to the project in such areas as developing project materials, participating in working group sessions, managing both data collection, and other research administrative tasks. TO, MB and TS will collaboratively finalize all materials. TS will have overarching responsibility for overseeing the developmental appropriateness of project activities. TS has specific knowledge, expertise in the area of sex and gender in development and intervention and will be primarily responsible for maintaining this perspective throughout the project period. TO, MB and TS will all contribute to the implementation of the study, analysis of data and report/article writing/publication. TO, MB and TS will all participate in community outreach, conference presentation and local stakeholder meetings.

TO, MB and TS will donate time from their institutionally based research time as needed (up to 20%). Based on our prior experience with implementation and effectiveness studies, we assess the time allotted in this application to be sufficient to meet project needs and have weighed this need in relation to our other teaching and research activities.

Collaboration with researchers outside of the project group. TO, MB and TS have a broad and well-established network of research colleagues both internal and external to their own organizations. TO is Chair of the research group Implementation- and intervention research (IIF-gruppen) at the Department of Social Work, University of Gothenburg. Participants include both senior and junior researchers both internal and external to the Department of Social Work and GU as well as practitioners and representatives from State organizations (e.g., National Board of Health and Welfare). TO is also a member of the Applied Developmental Science research group at the University of Stockholm’s Department of Psychology and the Network for Collaborative Research at the University of Borås. TO, MB and TS also collaborate with researchers outside of Sweden interested in youth transitioning from societal care. This Collaboration has led, for example to collaborative projects and publications (e.g., the Regional Research Institute for Human Services at Portland State University).

Relevence in relation to societal needs

This project has a strong focus on advancing client centered research and practice as relates to youth in societal care. Today, there are a large number of youth in societal care who do not have a parent or relative in Sweden which means they are fully fostered by the social welfare system. Without strengthening the services provided to these youth, many of them will languish through life and not reach their full potential or find their basic needs fulfilled as described in Swedish law (e.g., The Social Services Act; The Code Relating to Parenting and Guardianship; Convention on the Rights of the Child). In order to do this, we propose a strong and ongoing collaboration between our research group, social service practitioners, and youth. The project proposed in this application has been initiated by two separate client serving organizations (Farsta and SOS). This project aims to assess the effectiveness of an intervention (MVMV) developed in collaboration with practitioners and SOS developed by an NGO in light of various characteristics among the youth population served. Thereby advancing what we know about interventions for this vulnerable group and one of the municipal social services core populations. Additionally, there is a need for research on how organizations might transfer knowledge to practice. This project includes a study on a specific “implementation intervention” for knowledge transfer and application with the goal of assessing how these activities might ultimately impact service users. As such, this project has a strong service user perspective and will hopefully be of help in the strive to close the quality chasm, i.e. the gap between researchers and research on the one hand and pratitioners and practice on the other.

Utilization and communication of research results

A main goal of the current research program is to provide social service agencies in Sweden with an effective and feasible intervention that targets youth transitioning between societal care and independent living that can be sustainable over time. Thus, the research program has in its design to produce knowledge and tools, related to both methods and sustainable implementation, which can be of direct practical use in society. In fact, sustainable

use of the intervention in regular services is one of our research questions. We bridge the gap between research and practice via close collaboration with practitioners from social service organizations throughout the project period. The intervention and the implementation strategies will be developed together with the practitioners and will be informed by what matters in “the real world”. Thus, the results of the study will be very useful for the social services in all municipalities in Sweden. We will communicate our findings to practitioners and scholars. All collaborators, including the participating organizations, will gain knowledge about the results from the project. We will make sure to present information about our findings to agencies in a “practice-friendly” manner. We will also attend conferences targeted to professionals in the area and present the project and findings. We will also write short popular articles in journals for professionals. In terms of communication to the scientific community, our main modus of communication will be to publish research papers in international peer reviewed journals with open access. We will present the findings at international, scientific conferences (e.g., European Society for Prevention Research, Society for Prevention Research) in the social work and psychology fields. Finally, we will talk about the project in our teaching with students in social work and clinical psychology both in Gothenburg and in Lund. It is likely (and we will encourage) that students will write bachelor and master theses based on the data collected in the research program.

**References**

1. Vinnerljung B, Forsman H, Jacobsen H, Kling S, Kornør H, Lehmann S. Barn kan inte vänta. Stockholm: Nordens Välfärdscenter; 2015.

2. Vinnerljung B, Hjern A. Health care in Europe for children in societal out-of-home care. London: MOCHA/Imperial College; 2018.

3. Forsman H. Exploring educational pathways over the life course in children with out-of-home care experience: a multi-group path analysis. Children and Youth Services Review. 2020;11(104852).

4. Sallnäs M, Vinnerljung B. Instabilitet i familjehemsvården - en regional studie om sammanbrott bland yngre barn och land barn i långvarit famijehemsvård. Oplanerade avbrott i familjehemsplaceringar av yngre barn och långvarigt placerade barn. Stockholm: Socialstyrelsen; 2012. p. 24-68.

5. Brännström L, Vinnerljung B, Hjern A. Risk factors for teenage births: are they the same for foster youths as for majority population peers? Children and Youth Services Review. 2015;54:393-410.

6. von Borczykowski A, Vinnerljung B, Hjern A. Alcohol and drug abuse among young adults who grew up in substitute care - findings from a Swedish national cohort study. Children and Youth Services REview. 2013;35:1954-61.

7. Vinnerljung B, Hjern A. Cognitive, educational and self-support outcomes of long-term foster care versus adoption. A Swedish national cohort study. Children and Youth Services Review. 2011;33:1902 - 10.

8. Österberg T, Gustafsson B, Vinnerljung B. Children in out-of-home care and adult labor-market attachment: a Swedsih national register study. Journal of Public Child Welfare. 2016;10:414 - 33.

9. Bergström M, Cederblad M, Håkansson K, Jonsson AK, Munthe C, Vinnerljung B, et al. Interventions in foster family care: a systematic review. Research on Social Work Practice. 2020;30(1):3-18.

10. Yelick A. Research review: independent living programmes: the influence on youth ageing out of care (YAO). Child & Family Social Work. 2017;22:515-26.

11. SBU. Stöd till unga som ska flytta från placering i social dygnsvård: en systematisk översikt. Stockholm: Statens beredning för medicinsk och social utvärdering; 2020 2020-09-17. Report No.: 316 Contract No.: SBU 2019/513.

12. Greeson JKP, Garcia AR, Tan F, Chacon A, Ortiz AJ. Interventions for youth aging out of foster care: a state of the science review. Children and Youth Services Review. 2020;113:105005.

13. Catalano RF, Berglund ML, Ryan JAM, Lonczak HS, Hawkins DJ. Positive youth development in the United States: Research findings on evaluations of positive youth development programs. The ANNALS of the American Academy of Political and Social Science. 2004;591:98-124.

14. Ryan RM, Deci EL. Self-Determination Theory: Basic Psychological Needs in Motivation, Development and Wellness. New York: Guilford Press; 2017.

15. Lee J, Powers LE, Geenen S, Schmidt J, Blakeslee J, Hwant I. Mental health outcomes among youth in foster care with disabilities. Children and Youth Services Review. 2018;94:27-34.

16. Powers LE, Fullerton A, Schmidt J, Geenen S, Oberweiser-Kennedy M, Dohn J, et al. Perspectives of youth in foster care on essential ingredients for promoting self-determination and successful transition to adult life: My Life model. Children and Youth Services Review. 2018;86:277-86.

17. Fraser MW, Richman JM, Galinsky MJ, Day SH. Intervention Research: Developing Social Programs. Tripodi T, editor. New York: Oxford University Press; 2009.

18. Michie S, Atkins L, West R. The Behavior Change Wheel: A Guide to Designing Interventions. Surrey, UK: Silverback Publishing; 2014.

19. Carey RN, Connell LE, Johnston M, Rothman AJ, de Bruin M, Kelly MP, et al. Behavior change techniques and their mechanisms of action: A synthesis of links described in published intervention literature. Annals of Behavioral Medicine. 2019;53(8):693-707.

20. Michie S, West R, Campbell R, J. B, Gainforth H. ABC of Behaviour Change Theories. Great Britain: Silverback Publishing; 2014.

21. Hagger MS, Moyers S, McAnnally K, McKinley LE. Known knowns and known unknowns on behavior change interventions and mechanisms of action. Health Psychology Review. 2020;14(1):199-212.

22. Wilson TE, Hennessy EA, Falzon L, Boyd R, Kronish IM, Birk JL. Effectiveness of interventions targeting self-regulation to improve adherence to chronic disease medications: A meta-review of meta-analyses. Health Psychology Review. 2020;14(1):Online First.

23. Suls J, Mogavero JN, Falzon L, Pescatello LS, Hennessy EA, Davidson KW. Health behavior change in cardiovascular disease prevention and management: Meta-review of behaivor chabge techbiques to affect self-regulation. Health Psychology Review. 2020;14(1):online first.

24. Hennessy EA, Johnson BT, Acabchuk RL, McCloskey K, Stewart-James J. Self-regulation mechanisms in health behavior change: A systematic meta-review of meta-analyses, 2006-2017. Health Psychology Review. 2020;14(1):Online First.

25. Fixsen DL, Naoom SF, Blase KA, Friedman RM, Wallace F. Implementation Research: A Synthesis of the Literature. Tampa, FL; 2005.

26. Kaunitz C. Aggression Replacement Training (ART) i Sverige: spridning, programtrohet, målgrupp och utvärderin. Stockholm, Sweden: University of Stockholm; 2017.

27. Nilsen P. Making sense of implementaiton theories, models and frameworks. Implementation Science. 2015;10(53).

28. Chinman M, Imm P, Wandersman A. Getting to Outcomes 2004: Promoting Accountability Through Methods and Tools for Planning, Implementaion, and Evaluation. Santa Monica, CA: RAND Corporation; 2004.

29. Smelson DA, Chinman M, McCarthy S, Hannah G, Sawh L, Glickman M. A cluster randomized hybrid type III trial testing an implementaiton support strategy to facilitat the use of an evidence/based practice in VA homeless programs. Implementation Science. 2015;10.

30. Chinman M, Ebener P, Malone PS, Cannon J, d’Amico EJ, Acosta J. Testing implementation support for evidence-based programs in community settings: a replication cluster-randomized trial of Getting to Outcomes. Implementation Science. 2018;13.

31. Vinnerljung B, Andreassen T. Forskning i korthet: barn och unga i samhällets vård - forskning om den sociala dygnsvården. Stockholm; 2015.

32. Cohen J. Statistical Power Analysis for the Behavioral Sciences (2nd ed). Hillsdale, NJ: Erlbaum; 1988.

33. Olsson TM, Blakeslee J, Bergström M, Skoog T. Exploring fit for the cultural adaptation of a self-determination model for youth transitioning from out-of-home care: a comparison of a sample of Swedish youth with two samples of American youth in out-of-home caree. Children and Youth Services Review. 2020;119:Online First.

**Figure 1. Mitt val-min väg! Logic model**

**
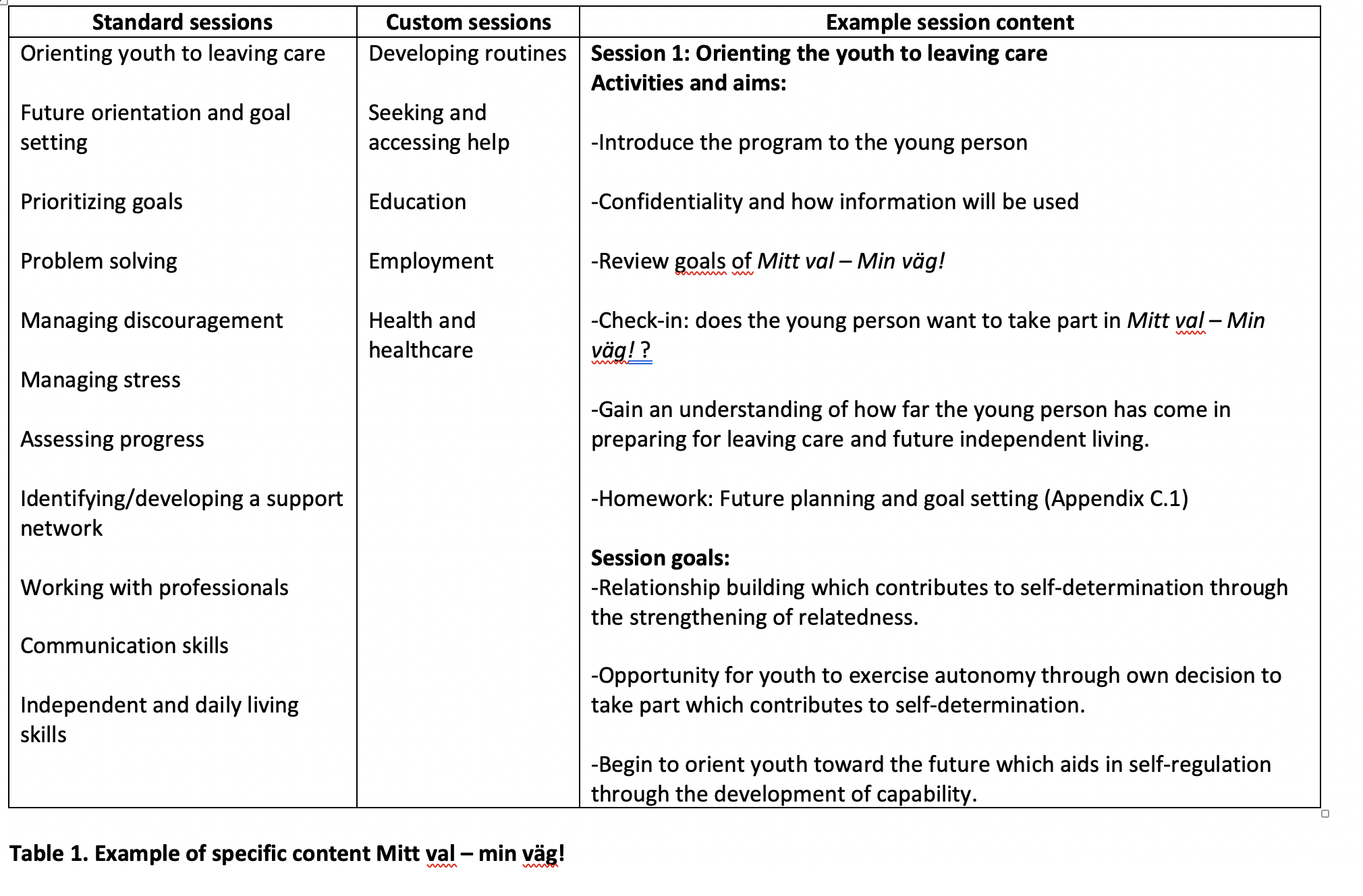
**


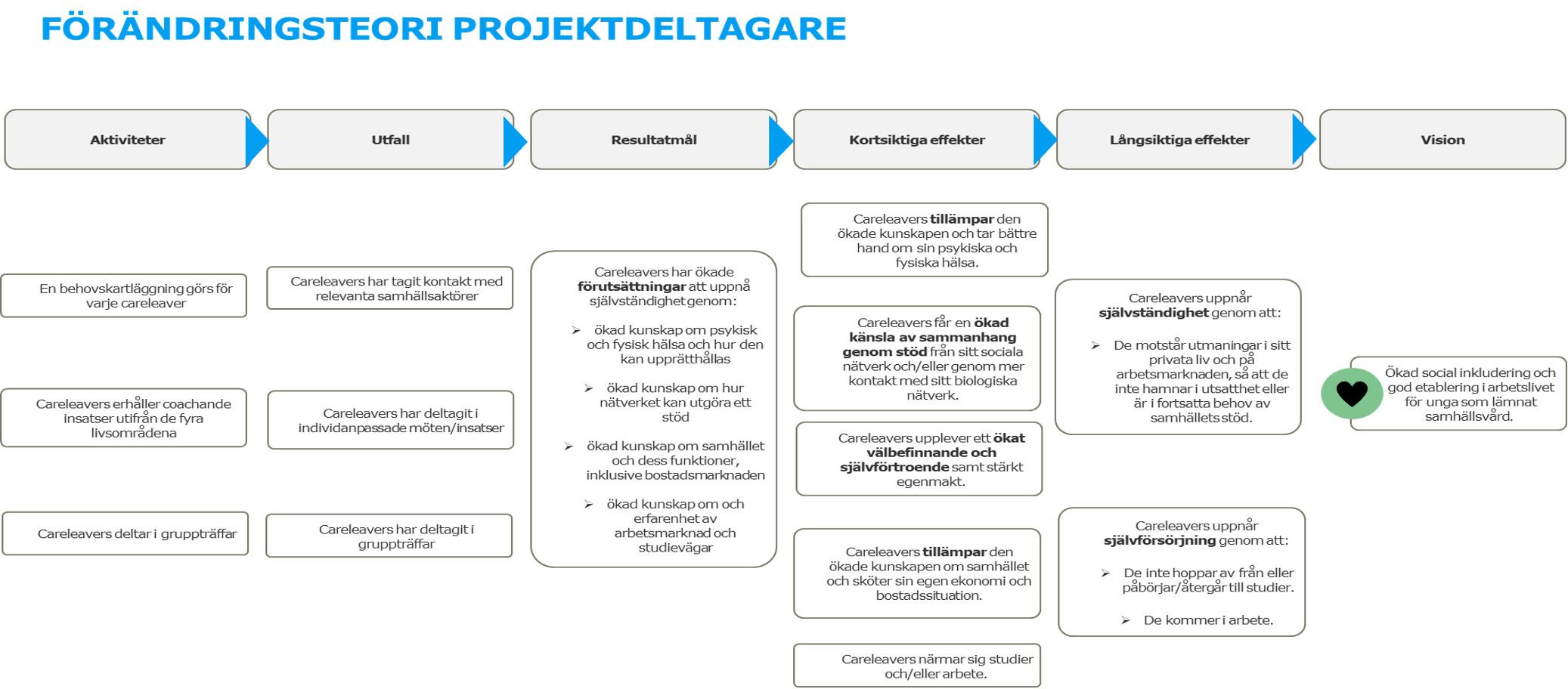


**Figure 2. Ungdomsprogram Logic model**
