# Supplementary material for: Study protocol for leaving care—A comparison study of implementation, change mechanisms and effectiveness of transition services for youth
Source: PLoS One. 2024 Feb 8;19(2):e0293952. doi: 10.1371/journal.pone.0293952 (PMC10852220; doi:10.1371/journal.pone.0293952)
Supplement: S2 File — (DOCX) [file pone.0293952.s003.docx]

Dnr 2022-02556-01

Linköping department other

DECISION

2022-08-23

Applicant research principal

University of Gothenburg

Researchers conducting the project

Tina Olsson

Project title

Leaving Care - a comparative study of implementation, change mechanisms, and effects.

of transition interventions for young people leaving community care

Information about the application

The application was submitted to the Swedish Ethical Review Authority 2022-05-04 and became valid 2022-05-10. The application is

previously dealt with at a meeting on 2022-05-31. A supplement requested by the Authority according to the

decision received 2022-07-06.

The Ethical Review Authority decides as follows.

DECISION

The Ethical Review Authority approves the research stated in the application, with the following conditions:

1. develop a consent form for therapists.

This decision can be appealed to the Board of Appeal for Ethical Review. How to appeal

can be found in the attached instructions.

2022-08-30

2022-02556-01-302159

Ethical Review Authority

Dnr 2022-02556-01

Linköping department other

On behalf of the Ethical Review Authority

Owe Horned

Chairperson

The decision has been made by the following persons:

Chairman

Owe Horned (former magistrate)

Members with scientific competence

Kristina Gustafsson (social work, ethnology, migration and integration studies, scientific

secretary)

Susanne Severinsson (pedagogy, social work, special education, social vulnerability and schooling,

scientific secretary)

Mikael Heimann (developmental psychology, rapporteur)

Annika Andersson (language learning, specifically second-language learning, psycholinguistics, cognitive neuroscience, educational psychology)

Gerhard Andersson (clinical psychology)

Motzi Eklöf (Health and Society/History of Nursing and Medicine)

Kristiina Heikkilä (elderly care, nursing science)

Dick Magnusson (technology and social change, community planning, social science, energy system

energy system studies)

Ulf Melin (informatics)

Members representing public interests

Peter Freij

Kristina Nero

Patrik Westlund

Erik Wågman

The decision is sent to

Responsible researcher: Tina Olsson

Representative of the research principal: Torun Österberg

2022-08-30

2022-02556-01-302159

Ethical Review Authority

Dnr 2022-02556-01

Linköping department other

How to appeal the Ethical Review Authority's decision

Who can appeal?

It is the research principal who may appeal the Ethical Review Authority's decision if it has been

gone against the applicant. The appeal must be in writing. The letter must be signed by

an authorized representative of the principal investigator.

If the researcher appeals, a power of attorney from the research principal must be attached.

When must the decision be appealed at the latest?

The appeal must be received by the Ethical Review Authority within three weeks of the date on which

the research principal received the decision.

What should the appeal contain?

The appeal must contain information about

1. the appellant's name, personal or organization number, address, telephone number and email address.

2. the decision being appealed (date of decision, project title and registration number).

3. how you think the Authority's decision should be changed and the reasons why the decision should be changed.

Where should the appeal be sent?

The appeal should be addressed to the Ethics Review Board of Appeal. But it should be sent or

submitted to the Ethics Review Authority.

If the appeal has been received in time, the Authority will forward the appeal and the

documents to the Ethics Review Board of Appeal.
